# Modeling the Chronotropic Effect of Isoprenaline on Rabbit Sinoatrial Node

**DOI:** 10.3389/fphys.2012.00241

**Published:** 2012-07-09

**Authors:** Henggui Zhang, Timothy Butters, Ismail Adeniran, Jonathan Higham, Arun V. Holden, Mark R. Boyett, Jules C. Hancox

**Affiliations:** ^1^Biological Physics Group, School of Physics and Astronomy, University of ManchesterManchester, UK; ^2^School of Computer Science and Technology, Harbin Institute of TechnologyHarbin, China; ^3^Multidisciplinary Cardiovascular Research Centre, Institute for Membrane and Systems Biology, University of LeedsLeeds, UK; ^4^Faculty of Medical and Human Sciences, University of ManchesterManchester, UK; ^5^School of Physiology and Pharmacology and Cardiovascular Research Laboratories, Medical Sciences Building, University WalkBristol, UK

**Keywords:** sinoatrial node, isoprenaline, action potential

## Abstract

**Introduction:** β-adrenergic stimulation increases the heart rate by accelerating the electrical activity of the pacemaker of the heart, the sinoatrial node (SAN). Ionic mechanisms underlying the actions of β-adrenergic stimulation are not yet fully understood. Isoprenaline (ISO), a β-adrenoceptor agonist, shifts voltage-dependent *I*_f_ activation to more positive potentials resulting in an increase of *I*_f_, which has been suggested to be the main mechanism underlying the effect of β-adrenergic stimulation. However, ISO has been found to increase the firing rate of rabbit SAN cells when *I*_f_ is blocked. ISO also increases *I*_CaL_, *I*_st_, *I*_Kr_, and *I*_Ks_; and shifts the activation of *I*_Kr_ to more negative potentials and increases the rate of its deactivation. ISO has also been reported to increase the intracellular Ca^2+^ transient, which can contribute to chronotropy by modulating the “Ca^2+^ clock.” The aim of this study was to analyze the ionic mechanisms underlying the positive chronotropy of β-adrenergic stimulation using two distinct and well established computational models of the electrical activity of rabbit SAN cells. **Methods and results:** We modified the Boyett et al. (2001) and Kurata et al. (2008) models of electrical activity for the central and peripheral rabbit SAN cells by incorporating equations for the known dose-dependent actions of ISO on various ionic channel currents (*I*_CaL_, *I*_st_, *I*_Kr_, and *I*_Ks_), kinetics of *I*_Kr_ and *I*_f_, and the intracellular Ca^2+^ transient. These equations were constructed from experimental data. To investigate the ionic basis of the effects of ISO, we simulated the chronotropic effect of a range of ISO concentrations when ISO exerted all its actions or just a subset of them. **Conclusion:** In both the Boyett et al. and Kurata et al. SAN models, the chronotropic effect of ISO was found to result from an integrated action of ISO on *I*_CaL_, *I*_f_, *I*_st_, *I*_Kr_, and *I*_Ks_, among which an increase in the rate of deactivation of *I*_Kr_ plays a prominent role, though the effect of ISO on *I*_f_ and [Ca^2+^]_i_ also plays a role.

## Introduction

β-Adrenergic stimulation increases the heart rate through accelerating the spontaneous activity of the pacemaker of the heart, the sinoatrial node (SAN; Abramochkin et al., 2009). It is believed that this occurs through β-adrenoceptor mediated modulation of ionic currents that contribute to pacemaker activity; however the precise ionic mechanisms underlying the effect of β-adrenergic stimulation are not yet fully elucidated. Experiments have shown that isoprenaline (ISO), a β-adrenergic agonist, increases the L-type calcium current (*I*_CaL_; Noma et al., 1980; Walsh et al., 1988; Zaza et al., 1996; Vinogradova et al., 2002; Ke et al., 2007; Alig et al., 2009), delayed rectifier potassium current (both *I*_Kr_ and *I*_Ks_; Walsh et al., 1988; Duchatelle-Gourdon et al., 1989; Giles et al., 1989; Yazawa and Kameyama, 1990; Freeman and Kass, 1993;Lei et al., 2000, 2002; Ke et al., 2007; Vinogradova et al., 2008), and shifts voltage-dependent activation of the hyperpolarization activated current (*I*_f_) toward positive potentials resulting in an increase of *I*_f_ (Brown et al., 1979; Cai et al., 1995; DiFrancesco, 1995; Zaza et al., 1996; Accili et al., 1997a,b; Bucchi et al., 2003; Barbuti et al., 2007; Alig et al., 2009; Baruscotti et al., 2010; Liao et al., 2010). As *I*_f_ has been regarded to be a major pacemaker current in mammalian pacemaker cells, an increase in *I*_f_ has been suggested to be the main mechanism underlying the positive chronotropic effect of β-adrenergic stimulation (DiFrancesco, 1995, 2010; Zaza et al., 1996; DiFrancesco and Borer, 2007; Liao et al., 2010). However, ISO has been found to increase the firing rate of the rabbit SAN when *I*_f_ was blocked by Cs^+^ (Cai et al., 1995), suggesting that *I*_f_ enhancement may not be predominantly responsible for the positive chronotropic action of ISO. Previous studies have shown that ISO increases the amplitude of the systolic rise of intracellular Ca^2+^ concentration ([Ca^2+^]_i_) in cardiac cells (Ju and Allen, 1999; Huser et al., 2000; Shannon et al., 2004; Maltsev and Lakatta, 2009; Wu et al., 2009). This raises the possibility that changes to [Ca^2+^]_i_ with ISO might contribute to the increase in firing rate via the “Ca^2+^ clock” mechanism (Vinogradova et al., 2002, 2008; Maltsev and Lakatta, 2009). A number of studies have shown that interventions altering [Ca^2+^]_i_ change the firing rate of pacemaker cells (Hagiwara, 1989; Ju and Allen, 1999; Huser et al., 2000; Shannon et al., 2004; Vinogradova et al., 2008; Maltsev and Lakatta, 2009; Wu et al., 2009). In both mammalian cardiac cells (Hagiwara, 1989; Huser et al., 2000; Vinogradova et al., 2002, 2008; Maltsev and Lakatta, 2009; Wu et al., 2009) and amphibian pacemaker cells in which *I*_f_ is absent (Ju and Allen, 1999), it has been found that the firing rate was dependent on the amplitude of the [Ca^2+^]_i_ transient: agents modifying SR Ca^2+^ release consequently affect the firing rate. β-Adrenergic stimulation increases the amplitude of the [Ca^2+^]_i_ transient. It has been argued that much of the increase in firing rate caused by β-stimulation seems to occur through the “Ca^2+^ clock” mechanism (Vinogradova et al., 2002, 2008; Maltsev and Lakatta, 2009). ISO has also been reported to increase the amplitude of the inward sustained current (*I*_st_; Guo et al., 1995, 1997; Shinagawa and Noma, 2000; Toyoda et al., 2005), and the amplitude of the *I*_Kr_ and *I*_Ks_ (Lei et al., 2000, 2002). It has also been found to shift the activation curve of *I*_Kr_ toward more negative membrane potentials and to increase its rate of deactivation (Yazawa and Kameyama, 1990; Lei et al., 2000, 2002; Ke et al., 2007). As *I*_st_ is activated over a membrane potential range between −70 and −50 mV (Guo et al., 1995, 1997; Shinagawa and Noma, 2000), increases in *I*_st_ might play a role in accelerating the rate of pacemaker activity. An increase in the rate of deactivation of *I*_Kr_ decreases the outward current, resulting in a relatively larger net inward current during diastolic depolarization, which could also contribute to the increase of the rate of spontaneous activity. In this study, we modified existing models of the electrical activity of central and peripheral rabbit SAN cells (Boyett et al., 2001; Kurata et al., 2008) in order to simulate the positive chronotropic effect of ISO and study the underlying ionic basis for the actions of ISO using a computational approach. We have incorporated equations for concentration-dependent actions of ISO on the macroscopic conductance of *I*_CaL_, *I*_Kr_ and *I*_Ks_, *I*_st_ and to describe changes in the kinetics of *I*_f_ and *I*_Kr_, and in [Ca^2+^]_i_. These equations have been constructed from experimental data. Using the models, the simulated effect of ISO shows concentration-dependence, which is quantitatively consistent with experimental recordings. To investigate the underlying ionic basis the effect of ISO, we examined its effects over a range of concentrations on the currents responsible for the pacemaker activity of the SAN, and we have investigated the individual role of actions of ISO on currents of *I*_CaL_, *I*_f_, *I*_Kr_, *I*_st_, and [Ca^2+^]_i_. We found that the chronotropic effect of ISO reflects integrated actions on ionic currents *I*_CaL_, *I*_f_, *I*_Kr_, *I*_Ks_, *I*_st_, and [Ca^2+^]_i_, amongst which the increase in the rate of deactivation of *I*_Kr_ appears to be particularly important, whilst the effect of ISO on *I*_f_ and [Ca^2+^]_i_ also plays a role.

## Model Development

Based on the voltage clamp experimental data on the kinetics of ionic channels and regional differences in the ionic current densities, well established action potentials models of the center and periphery of the rabbit SAN cells have been developed (Zhang et al., 2000; Boyett et al., 2001; Kurata et al., 2008). The Zhang et al. (2000) models were upgraded by incorporating an inward sustained current (*I*_st_) and intracellular Ca^2+^ regulation equations (Boyett et al., 2001). In this study, we modified the models of Boyett et al. (2001) and Kurata et al. (2008) by including equations describing the actions of ISO on *I*_CaL_, *I*_f_, *I*_Kr_, *I*_Ks_, *I*_st_, and [Ca^2+^]_i_.

### Increase of *I*_CaL_

Isoprenaline was found, experimentally, to increase *I*_CaL_ without a significant change in its kinetics (Zaza et al., 1996). The increase in *I*_CaL_ is dose dependent. The equation describing the dose-dependent increase of *I*_CaL_ was constructed from experimental data of Zaza et al. (1996) obtained from rabbit SAN cells, which take the form:

(1) $$f_{\text{Ca}}=f_{\text{Ca},\max}\frac{\left[\text{ISO}\right]}{K_{0.5,\text{Ca}}+\left[\text{ISO}\right]}$$

**Figure 1:**
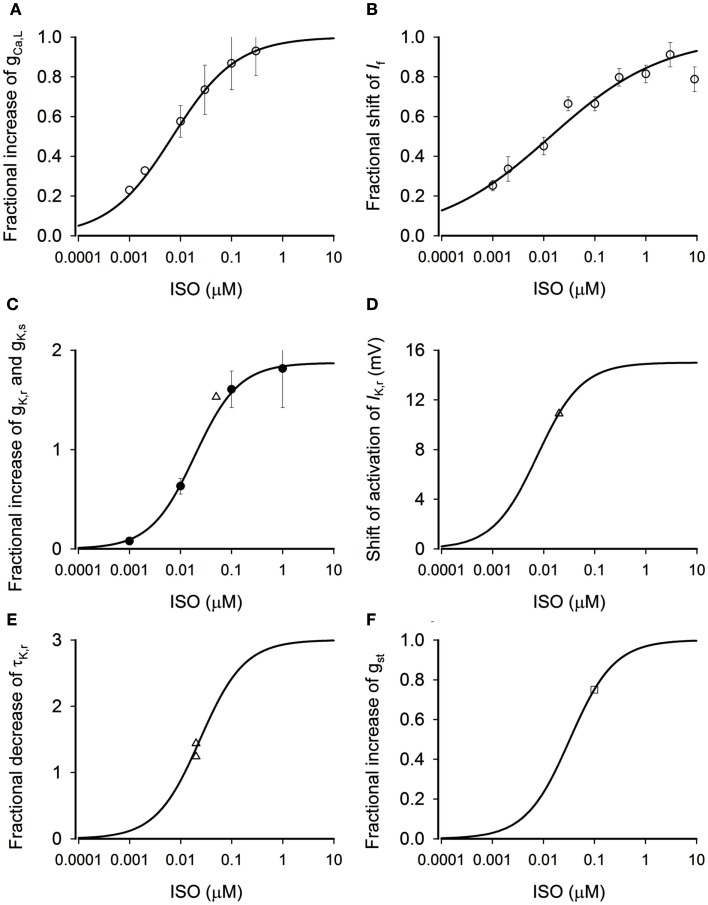
**Concentration-dependent effects of ISO**. **(A)** Dose-dependent fractional increase of *g*_CaL_. Open circles: experimental data from Zaza et al. (1996) from rabbit SAN cells. Solid line: computed from model. **(B)** Dose-dependent fractional shift of the *I*_f_ activation curve. Open circles: data from Zaza et al. (1996) from rabbit SAN cells. Solid line: computed from model. **(C)** Dose-dependent fractional increase of *g*_Kr_. Solid circles: data from Yazawa and Kameyama (1990) from guinea-pig SAN cells. Open triangle: data from Lei et al. (2000) from rabbit SAN cells. Solid line: computed from model. **(D)** Dose-dependent shift of *I*_Kr_ activation curve. Open triangle: data from Lei et al. (2000) from rabbit SAN cells. Solid line: computed from model. **(E)** Dose-dependent fractional decrease of time constant of deactivation of *I*_Kr_. Open triangles: data from Lei et al. (2000) from rabbit SAN cells. Solid line: computed from model. **(F)** Dose-dependent fractional increase of *g*_st_. Open square: data from Guo et al. (1995) from rabbit SAN cells. Solid line: computed from model.

Where *f*_Ca_ is the percentage of increase of *I*_CaL_. By fitting Eq. 1 to the data of Zaza et al. (1996; circles) and Vinogradova et al. (2002; triangles) as shown in Figure 1A, we obtained the best fit values for *f*_Ca,max_ (the maximum percentage increase *I*_CaL_) and *K*_0.5,Ca_ (the ISO concentration required to half-maximally increase of *I*_CaL_) as 0.54 (i.e., 54%) and 7 nM, respectively. The obtained *K*_0.5,Ca_ (7 nM) matches to the experimental data of EC_50_ of ISO on *I*_CaL_ (Zaza et al., 1996). The solid line in Figure 1A shows the relationship between the increase in *I*_CaL_ and the ISO concentration predicted by Eq. 1.

### Shift of the activation curve of *I_f_*

Isoprenaline increases *I*_f_ by shifting its voltage-dependent activation curve toward more positive membrane potentials (Zaza et al., 1996; Accili et al., 1997a,b). The equation to describe the shift, *S*_f_ (in mV), was constructed from the experimental data of Zaza et al. (1996) obtained from rabbit SAN cells as shown below:

(2) $$S_{\text{f}}=S_{\text{f},\max}\frac{{\left[\text{ISO}\right]}^{n_{\text{f}}}}{K_{0.5,\text{f}}^{n_{\text{f}}}+{\left[\text{ISO}\right]}^{n_{\text{f}}}}$$

By fitting the Eq. 2 to the data of Zaza et al. (1996; Figure 1B, circles), we obtained the best fit values for *S*_f,max_ (the maximum shift), *K*_0.5,f_ (the ISO concentration required to half-maximally shift the activation curve), and *n*_f_ (the Hill coefficient) as 9.62 mV, 13.5 nM, and 0.392, respectively, which are close to those obtained by Zaza et al. (1996). The solid line in Figure 1B shows the relationship between the shift of *I*_f_ activation curve and the ISO concentration predicted by Eq. 2.

### Actions on *I*_Kr_ and *I*_Ks_

Isoprenaline has complex actions on *I*_K_. β-adrenergic agonists have been reported to increase *I*_K_ in rabbit SAN cells (Lei et al., 2000, 2002), small multi-cellular preparations of rabbit SAN (Walsh et al., 1988), guinea-pig SAN cells (Freeman and Kass, 1993), guinea-pig ventricular myocytes (Yazawa and Kameyama, 1990), and frog atrial cells (Giles et al., 1989). In rabbit SAN cells, *I*_K_ consists of two components, a rapidly activating *I*_Kr_ and a slowly activating *I*_Ks_, of which *I*_Kr_ is normally the predominant component (Habuchi et al., 1995; Ito and Ono, 1995; Lei and Brown, 1996; Lei et al., 2000, 2002). ISO has been found to increase the current amplitude of *I*_Kr_ and *I*_Ks_ (Lei et al., 2000, 2002), increase the rate of deactivation of *I*_Kr_ in rabbit SAN cells (Lei et al., 2000; Ke et al., 2007), and shift the activation curve of *I*_Kr_ toward more negative membrane potentials in guinea-pig ventricular myocytes (Yazawa and Kameyama, 1990), frog atrial cells (Giles et al., 1989), and rabbit SAN cells (Lei et al., 2000).

### Increase of *g*_Kr_ and *g*_Ks_ (*I*_Kr_ and *I*_Ks_)

The equation for concentration-dependent increase in *g*_Kr_ was constructed from experimental data of Lei et al. (2000, 2002) obtained from rabbit SAN cells shown as in Figure 1C (triangles) and data of Yazawa and Kameyama (1990) obtained from guinea-pig ventricular myocytes shown as in Figure 1C (circles). The equation takes the form:

(3) $$f_{\text{K}}=f_{\text{K},\max}\frac{\left[\text{ISO}\right]}{K_{0.5,g_{\text{K}}}+\left[\text{ISO}\right]}$$

Where *f*_K_ is the percentage increase of *I*_Kr_. By fitting Eq. 3 to the experimental data of Lei et al. (2000, 2002) and Yazawa and Kameyama (1990), we obtained the best fit values for *f*_K,max_ (the maximum percentage increase *I*_K_) and *K*_0.5,Ca_ (the ISO concentration required to half-maximally increase of *g*_Kr_) as 1.87 (i.e., 187%) and 19 nM, respectively. The solid line in Figure 1C shows the relationship between the increase in *g*_Kr_ and the ISO concentration predicted by Eq. 3.

Isoprenaline has also been found to increase *I*_Ks_: experimental data have shown that 10 nM ISO increased *I*_Ks_ by about 20% (Lei et al., 2002), which is comparable to the increase of *I*_Kr_ with a similar ISO concentration. Due both to this similarity and to the lack of availability of complete experimental concentration-response data, in this study we assumed a similar concentration-dependent modulation by ISO of *g*_Ks_ and *g*_Kr_, both of which were modeled by Eq. 3.

### Shift in activation of *I*_Kr_

The equation to describe the shift in the voltage-dependent activation relation of *I*_Kr_ (*S*_K_) was constructed from experimental data of Lei et al. (2000) obtained from rabbit SAN cells. As there are not sufficient experimental data available in respect of concentration-dependence of the effect, we assumed that the concentration-dependent shift of the *I*_Kr_ activation curve is the same as dose-dependent increase of *g*_Kr_, which takes the form:

(4) $$S_{\text{K}}=S_{\text{K},\max}\frac{\text{[ISO]}}{K_{0.5,\text{Kacti}}+\left[\text{ISO}\right]}$$

By fitting Eq. 4 to the experimental data of Lei et al. (2000) as shown in Figure 1D (triangle), we obtained the best fit value of *S*_K,max_ (the maximal shift of *I*_K_, in mV) as −15 mV and *K*_0.5,Kacti_ as 7.5 nM.

### Increase in the rate of deactivation of *I*_Kr_

The equation describing the increase in the rate of deactivation of *I*_Kr_ was constructed from experimental data on *I*_K_ from Lei et al. (2000) obtained from rabbit SAN cells, shown in Figure 1E (triangles). In the Figure, data are presented as the decrease of the time constant of deactivation (τ_Kr_). Once again, we assumed that the dose-dependent change of τ_Kr_ is as the same as the dose-dependent increase of *g*_Kr_. The equation takes the form:

(5) $$d_{\tau_{\text{Kr}}}=d_{\tau_{\text{Kr}},\text{max}}\frac{\left[\text{ISO}\right]}{K_{0.5,\tau_{\text{Kr}}}+\left[\text{ISO}\right]}$$

$d_{\tau_{\text{Kr}}}$ is the fractional decrease of τ_Kr_. By fitting the equation to the experimental data of Lei et al. (2000), we obtained the best fit value of $d_{\tau_{\text{Kr}},\text{max}}$ as 3.0 and *K*_0.5_, τ_Kr_ as 24 nM.

### Increase of *I*_st_

Isoprenaline has been reported to increase *I*_st_ (Guo et al., 1995, 1997; Shinagawa and Noma, 2000; Toyoda et al., 2005). The equation for the increase of *I*_st_ was constructed from experimental data obtained from rabbit SAN cells (Guo et al., 1997). Due to a lack of availability of sufficient experimental data, we assumed that the concentration-dependent increase of *I*_st_ is the same as the concentration-dependent increase of *g*_Kr_. The equation takes the form:

(6) $$f_{\text{st}}=f_{\text{st},\max}\frac{\left[\text{ISO}\right]}{K_{0.5,\text{st}}+\left[\text{ISO}\right]}$$

By fitting Eq. 6 to the experimental data of Guo et al. (1997) as shown in Figure 1F (open square), we obtained the best fit value of *f*_st,max_ as 1.0 and *K*_0.5,st_ as 33 nM.

### Ca^2+^ handling

Isoprenaline was found to increase the amplitude and minimal diastolic level of [Ca^2+^]_i_ in mammalian (Hagiwara, 1989; Huser et al., 2000; Vinogradova et al., 2002, 2008; Shannon et al., 2004; Maltsev and Lakatta, 2009; Wu et al., 2009) and amphibian (Ju and Allen, 1999) pacemaker cells. It has been found that ISO altered SR Ca^2+^ uptake and release by stimulation of calmodulin kinase II (CamKII; Shannon et al., 2005; Maltsev and Lakatta, 2009). In simulations of the effect of ISO, we adopted the approach of Kharche et al. (2011) to modify the Ca^2+^ handling equations to increase the amplitude and minimal diastolic level of [Ca]_i_, as observed in experimental studies (Ju and Allen, 1999; Vinogradova et al., 2002) by increasing the maximal SR Ca^2+^ release (by 20%) and reducing the SR Ca^2+^ release (by 20%) fluxes.

Equations 1–6 were incorporated into the action potential mathematical models of Boyett et al. (2001) and Kurata et al. (2008) for central and peripheral rabbit SAN cells to simulate the chronotropic effect of ISO and to investigate the ionic basis underlying such a chronotropic effect.

## Results

### Chronotropic effect of isoprenaline

**Figure 2:**
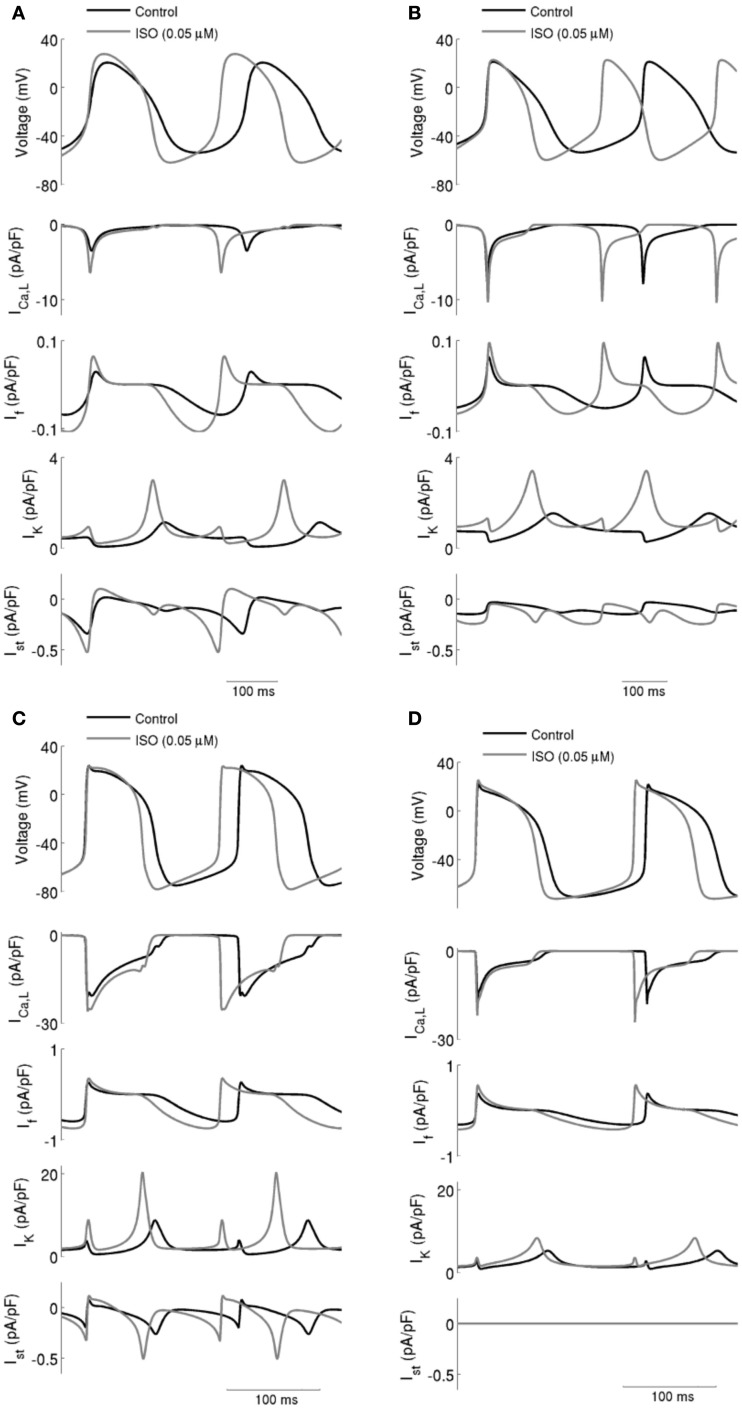
**I_CaL_, I_f_, I_K_ (sum of I_Kr_ and I_Ks_), and I_st_ during action potentials in control and with ISO**. Data in control and with 0.05 μM ISO are shown for: **(A)** Boyett et al.; **(B)** Kurata et al. central cell models. **(C)** Boyett et al.; **(D)** Kurata et al. peripheral cell models. In all four cell models, ISO caused an increase in spontaneous firing rate and this was accompanied by increases in *I*_CaL_, *I*_f_, *I*_K_, and *I*_st_.

The simulated effects of ISO on spontaneous action potentials are shown in Figure 2. In the figure, the left-hand panels show the results computed from the central (A) and peripheral (C) Boyett et al. cell models, the right-hand panels show the results computed from the central (B) and peripheral (D) Kurata et al. cell models. For each cell-type and each model, the effects of ISO with a concentration of 0.05 μM are shown. In all cell models, ISO affected the shape of action potentials: it accelerated the firing rate, shortened the action potential duration, increased the amplitude of action potentials, and the maximal diastolic potential (the maximal diastolic potential became more negative). These simulated effects of ISO on action potentials are consistent with experimental observations on rabbit SAN cells (Zaza et al., 1996; Lei et al., 2000).

Effects of ISO on ionic currents underlying action potentials (upper panel in each of Figure 2; Left panels for the Boyett et al. central (Figure 2A) and peripheral (Figure 2C) cell models; and right panels for the Kurata et al. central (Figure 2B) and peripheral (Figure 2D) of cell model were then studied [namely on *I*_CaL_, *I*_f_, *I*_K_ (the sum of *I*_Kr_ and *I*_Ks_), and *I*_st_]. The profiles of *I*_CaL_ (the second panel), *I*_f_ (the third panel), *I*_K_ (the fourth panel), and *I*_st_ (the fifth panel) are shown in Figure 2 under control conditions and in the presence of 0.05 μM ISO. In the simulated presence of ISO the inward currents *I*_CaL_, *I*_f_, and *I*_st_ were increased, while net *I*_K_ was increased in the repolarization phase but decreased in the diastolic depolarization phase. The increased *I*_K_ during action potential repolarization sped up the rate of repolarization (thereby shortening action potential duration) and increased the maximal diastolic potential. Increases of the inward currents *I*_CaL_, *I*_f_, and *I*_st_, together with a decrease of the outward current *I*_K_ in the depolarization phase accelerated the rate of depolarization and therefore the rate of the spontaneous activity. The increase in action potential amplitude can be attributed to an increase in *I*_CaL_.

**Figure 3:**
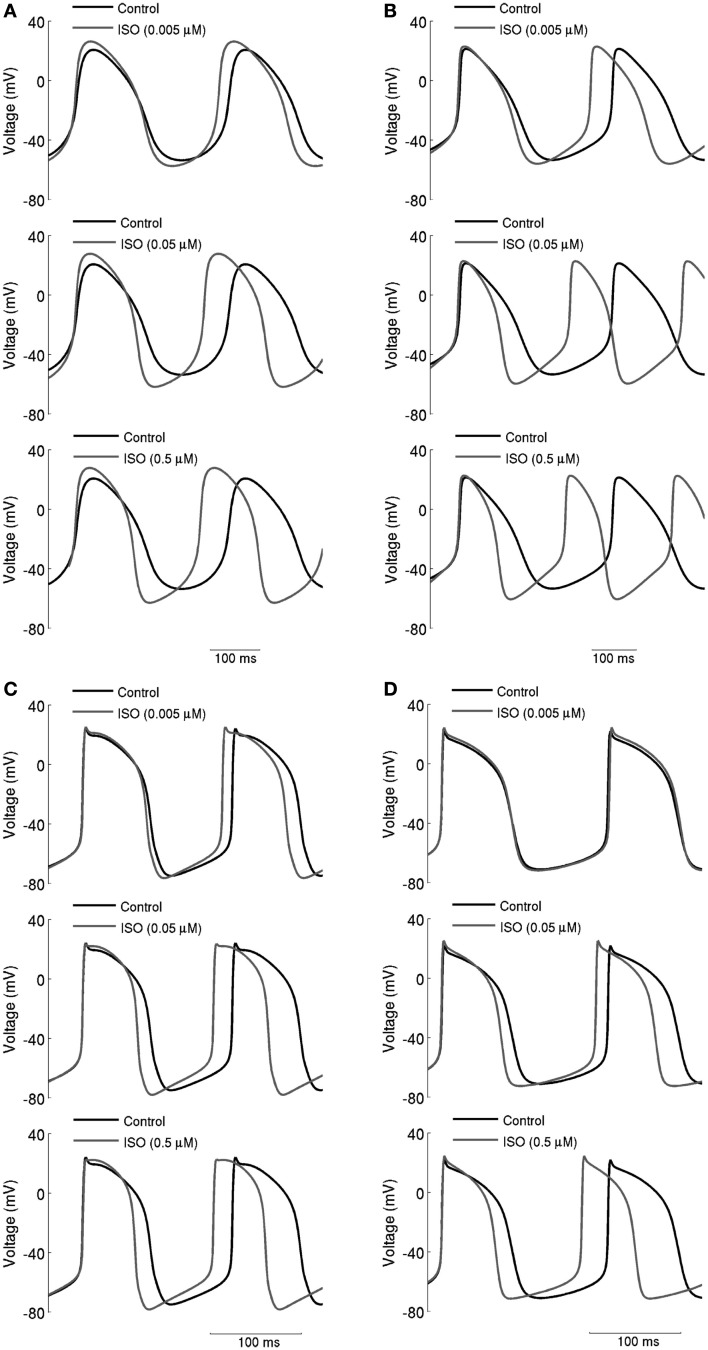
**Concentration-dependent effects of ISO on action potentials**. **(A)** Boyett et al.; **(B)** Kurata et al. central cell models. **(C)** Boyett et al.; **(D)** Kurata et al. peripheral cell models. Action potentials are shown under control conditions and in the presence 0.005, 0.05, and 0.5 μM ISO. Spontaneous firing rate was increased by ISO in a dose-dependent manner.

**Figure 4:**
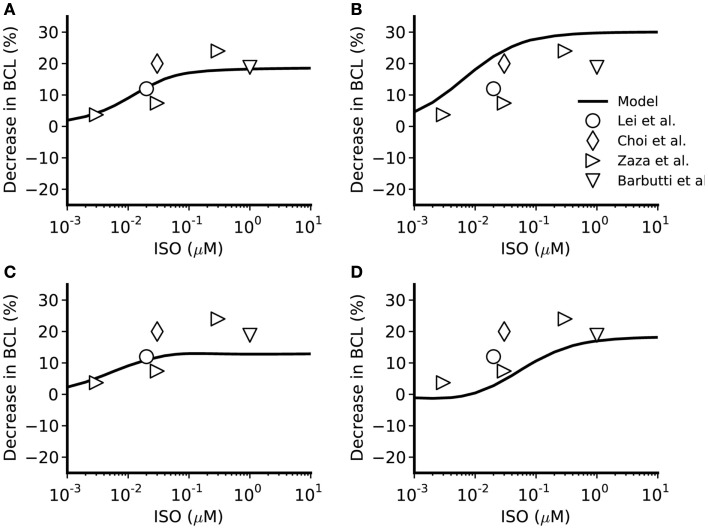
**Concentration-dependent percentage decrease of pacemaking cycle length (BCL) by ISO**. **(A,B)** Boyett et al. and Kurata et al. central cell models. **(C,D)** Boyett et al. and Kurata et al. peripheral cell models. The figure incorporates concentration-response data from the models and relevant experimental data. Black line: data from the standard configuration of model in which effects of ISO are incorporated for all of *I*_CaL_, *I*_f_, *I*_Kr_, *I*_Ks_, *I*_st_, and Ca^2+^ transient. Open circles: experimental data of Lei et al. (2000) from rabbit SAN cells. Open right-facing triangle: data of Zaza et al. (1996) from rabbit SAN cells. Open diamonds: data of Choi et al. (1999) from rabbit SAN cells. Open inverted triangles: data of Barbuti et al. (2007) from rabbit SAN cells.

**Figure 5:**
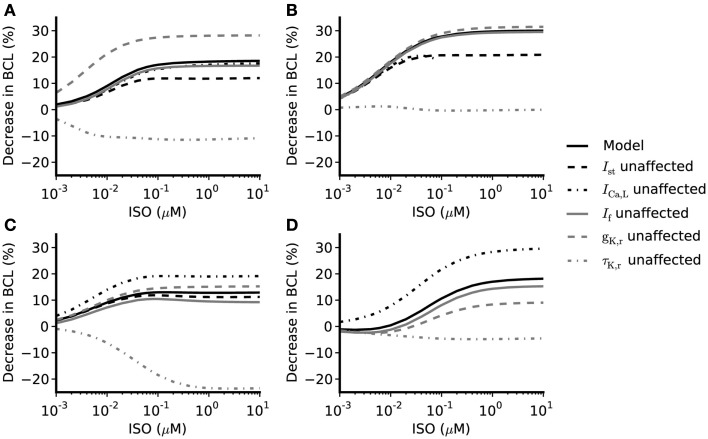
**Concentration-dependent percentage decrease of pacemaking cycle length (BCL) by ISO under different simulation conditions**. (**A,B**) Boyett et al. and Kurata et al. central cell models (**C,D**) Boyett et al. and Kurata et al. peripheral cell models. Black solid line: data from the standard configuration of model in which effects of ISO are incorporated for all of *I*_CaL_, *I*_f_, *I*_Kr_, *I*_Ks_, *I*_st_, and Ca^2+^ transient. Black dash-dotted line: data from model when effects on *I*_CaL_ omitted. Gray solid line: data from model when effects on *I*_f_ omitted. Gray dashed line: data from model when *g*_Kr_ not affected by ISO. Gray dash-dotted line: data from model when time constant of deactivation of τ_Kr_ not affected by ISO. Black dashed line: data from model when effects of ISO on *I*_st_ omitted.

The simulated effects of ISO exhibited clear concentration-dependence: the higher the simulated ISO concentrations, the greater the observed chronotropic effect. This is shown in Figure 3, in which action potentials computed under control conditions are superimposed on those computed with different ISO concentrations for the central (A) and peripheral (C) Boyett et al. cell models (left-hand panels), and the central (B) and peripheral (D) Kurata et al. (right-hand panels) cell models. In simulations using the Boyett et al. (2001) models, 0.005 μM ISO increased the rate by 7% for the central cell model and by 6% for the peripheral cell model. 0.05 μM ISO increased the rate by 20% for the central cell model and 15% for the peripheral cell model. 0.5 μM ISO increased the rate by 21% for the central cell model and 16% for the peripheral cell model. In simulations using the Kurata et al. (2008) models, 0.005 μM ISO increased the rate by 13% for the central cell model but did not significantly change rate for the peripheral cell model. 0.05 μM ISO increased the rate by 25% for the central cell model and 7% for the peripheral cell model; 0.5 μM ISO increased the rate by 29% for the central cell model and 16% for the peripheral cell model. The results summarizing the simulated chronotropic effects of ISO are shown in Figure 4, in which the percentage of decrease of pacemaking cycle length (BCL) is plotted against ISO concentration. The data computed from the central cell models of Boyett et al. and Kurata et al. are shown in Figures 4A,B, respectively. The data computed from the peripheral cell models of Boyett et al. and Kurata et al. are shown in Figures 4C,D, respectively. The simulated data (solid line) were compared with the experimental data obtained from rabbit isolated SAN cells by Lei et al. (2000; open circle), Zaza et al., 1996; open right-facing triangle), Choi et al. (1999; open diamond), and Barbuti et al. (2007; open inverted triangle). Experimentally, 0.02 μM ISO increased the rate by 13% (Lei et al., 2000), similar to simulated response of all four cell models to the same ISO concentration. The discrepancy between the simulations and the experimental data of Zaza et al. (1996) or the Choi et al. (1999) is slight and could be attributable to model limitations (see Discussion). Note that the simulation data from the central cell models matched better to experimental data than the peripheral cell models as in those experimental studies only primary SAN cells were considered without distinguish between central and peripheral cells. The roles of *I*_CaL_, *I*_f_, *I*_Kr_, *I*_st_, and [Ca^2+^]_i_ in the ISO-produced positive chronotropy were also considered, as shown in Figure 5.

### The role of *I*_CaL_

The role of *I*_CaL_ in ISO-induced positive chronotropy was investigated by comparing the effect of ISO when its action on *I*_CaL_ was incorporated into the models with that observed when this action was removed. The concentration-dependence of the change in rate (when *I*_CaL_ is not affected by ISO while *I*_f_, *I*_Kr_, *I*_Ks_, [Ca]_i_, and *I*_st_ are affected by ISO) is shown in Figure 5 (black dash-dotted line), whilst the standard response [obtained when all actions of ISO were considered (solid black line)] is also shown. In the central cell models, without affecting *I*_CaL_, the concentration-dependent change of rate was similar to the standard response. For the peripheral cell models, when *I*_CaL_ was unaltered by ISO, the concentration-dependent change of rate was found always to be larger than the standard value in the whole range of ISO concentration considered. This implies that the increase of *I*_CaL_ did not contribute to the ISO increase of the rate under our simulation conditions; indeed, if anything, it decreased rate.

### The role of *I*_f_

Isoprenaline shifts the voltage dependence of activation curve of *I*_f_ to more positive membrane potentials with a maximal shift of about +15 mV in rabbit SAN cells (Zaza et al., 1996; Accili et al., 1997a,b; and about +7 to +18 mV in murine SAN cells; Alig et al., 2009; Baruscotti et al., 2010; Liao et al., 2010). This shift results in an increase in *I*_f_ over voltages relevant to the pacemaker potential range. Adopting an approach similar to that taken above for *I*_CaL_, the role of *I*_f_ in ISO-induced positive chronotropy was investigated by comparing simulations incorporating the effects of ISO on *I*_f_ with those in which this action was not incorporated. The simulated data are shown in Figure 5 (solid gray line) and compared with the standard simulations (solid black line). In both the central and peripheral cell models the concentration-dependent changes of the rate were reduced compared with the standard value, which suggests *I*_f_ contributes to the ISO-induced positive chronotropy.

**Figure 6:**
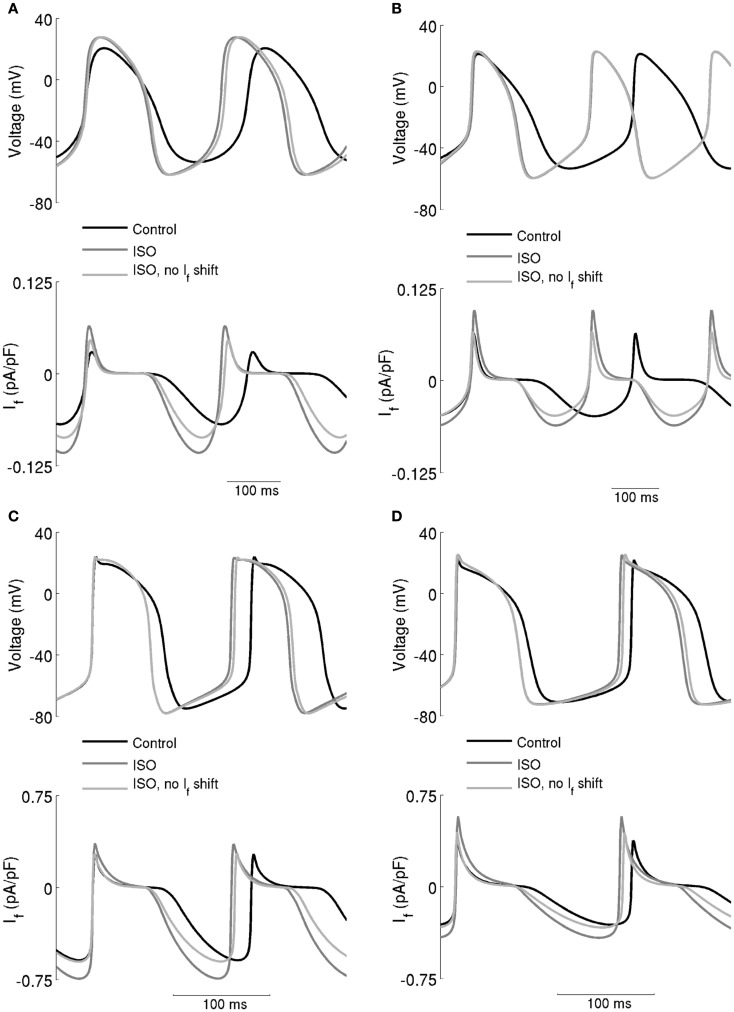
**Effect of the ISO-induced increase of *I*_f_ on spontaneous activity**. **(A)** Boyett et al. and **(B)** Kurata et al. central cell models. **(C)** Boyett et al. and **(D)** Kurata et al. cell models. Each panel shows action potentials (upper traces) and records of *I*_f_ (lower traces) under control conditions and in the presence of 0.05 μM ISO. Two simulations with ISO are shown – in one, *I*_f_ was affected and in the other it was not. This shows that the role of *I*_f_ in the chronotropic effect of ISO is small.

The computed action potentials and *I*_f_ under different conditions are presented in Figure 6, in order to illustrate more clearly the role of *I*_f_ in the chronotropic effect of ISO. The left-hand panels show the results computed from the Boyett et al. models, whilst the right-hand panels show the results computed from the Kurata et al. models. “control” traces were obtained without simulating effects of ISO, traces labeled “ISO” were obtained from incorporating all actions of 0.05 μM ISO, whilst traces labeled “ISO, no *I*_f_ shift” omit actions on *I*_f_. In these simulations, ISO accelerated the pacemaking rate by reducing the cycle length of spontaneous activity for both the central (Figures 6A,B) and peripheral (Figures 6C,D) cell models. In the case of “ISO, no *I*_f_ shift,” the pacemaker activities of both models are slowed down, but by less than 3% compared to the “ISO” condition. The positive shift in voltage-dependent activation for *I*_f_ generated a significant increase in the magnitude of *I*_f_ as shown in the lower panels in Figures 6A,B (central models) and Figures 6C,D (peripheral models). However, our simulations indicate that the increase of *I*_f_ was not exclusively the result of this shift, as when it was absent there was still an increase in *I*_f_, attributable to the increase of the maximal diastolic potential.

### The role of *I*_Kr_

The role of *I*_Kr_ in the chronotropic effect of ISO was comprised of three components: the increase of *g*_Kr_, the shift of the *I*_Kr_ activation curve, and the increase of the rate of deactivation of *I*_Kr_. By adopting a simulation approach, it was possible to investigate in turn the contribution of each of these elements to the action of ISO. The computed concentration-dependent changes in spontaneous rate of the central and peripheral models when the action of ISO on *g*_Kr_ was omitted are shown in Figure 5 (dashed gray line) and compared with the standard ISO simulation (solid black line). In these simulations, the Boyett et al. and the Kurata et al. central cell models responded in a qualitatively similar fashion. In both Boyett et al. and Kurata et al. central SAN models (Figures 5A,B), removing the ISO action on *g*_Kr_ accelerated pacemaking as demonstrated by a greater decrease in pacemaking cycle length as compared to the standard condition. Such an enhanced chronotropic effect without ISO modification of *g*_Kr_ was also observed in the Boyett et al. peripheral cell model (Figure 5C), though data from the Kurata et al. peripheral cell model (Figure 5D) showed a different response. In the Kurata et al. peripheral cell model, removing the ISO action on *g*_Kr_ resulted in a reduced decrease in the pacemaking cycle length as compared to the standard value.

In simulations, we also computed concentration-dependent change in spontaneous rate when the action of ISO on voltage dependence of activation of *I*_Kr_ was omitted, and compared the results with the standard value (data not shown). In both the Boyett et al. and Kurata et al. central cell models, the computed values are larger than the standard values, which suggests that the shift in voltage-dependent activation of *I*_Kr_ does not contribute to ISO-stimulated increase in pacemaking rate (in contrast, it appeared actually to reduce pacemaking rate). Omitting this action of ISO had a negligible effect on the response of the peripheral cell models. Similarly, the concentration-dependent change of the pacemaking rate when the action of ISO on τ_Kr_ was omitted is also shown in Figure 5 (gray dash-dotted line). In both central and peripheral cell models, removal of the action of ISO on τ_Kr_ had a dramatic influence of the chronotropic effect of ISO. In the central cell models, when effects on τ_Kr_ were omitted ISO decreased the pacemaking rate (more so in Boyett et al. model, compared to that of Kurata et al.). Similar effects were seen for the peripheral cell models, for which ISO significantly slowed down the pacemaking rate at high concentrations. The results of these simulations indicate that the effect of ISO on τ_Kr_ plays an important role in the chronotropic effect of ISO.

**Figure 7:**
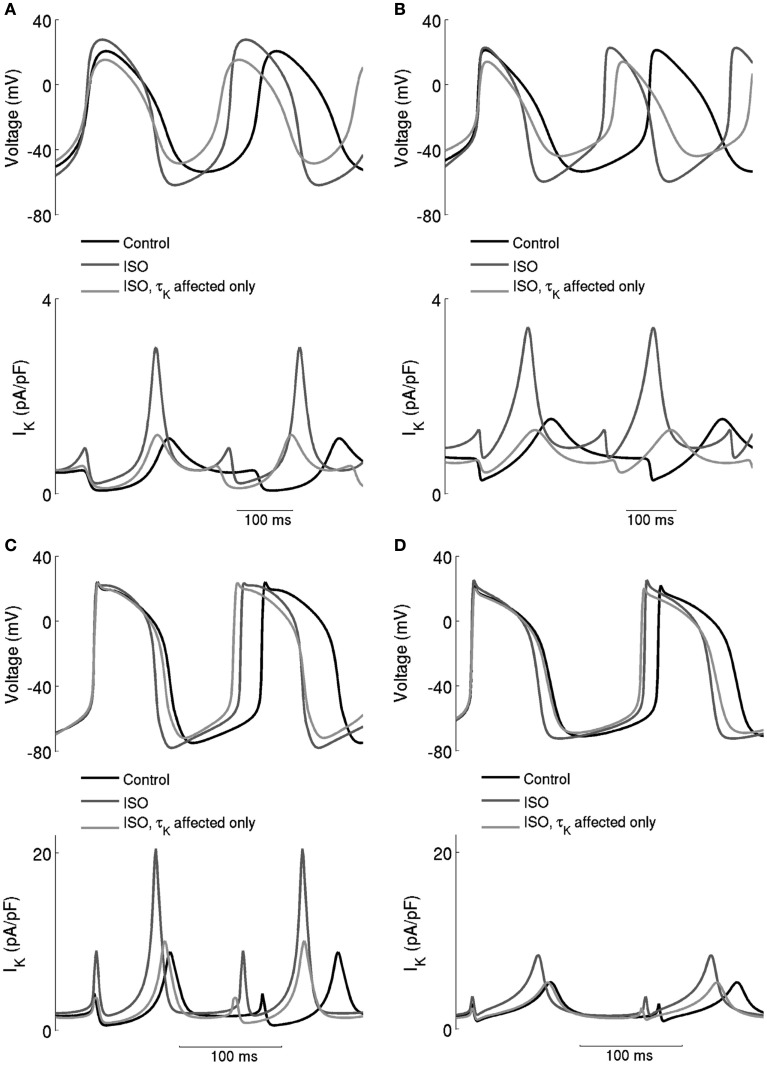
**Effects of ISO-induced increase of the time constant of deactivation of *I*_Kr_**. Spontaneous activity in the central **(A)** Boyett et al. **(B)** Kurata et al. cell models and peripheral **(C)** Boyett et al. **(D)** Kurata et al. cell models. Each panel shows action potentials and records of *I*_Kr_ under control conditions and in the presence of 0.05 μM ISO. Two simulation conditions with ISO are shown – in one, only *τ*_Kr_ was affected and in the other modulatory effects on currents were incorporated. When only *τ*_K_ was affected, there was a substantial increase of the firing rate, but there was also a large decrease of the maximum diastolic potential, which is not observed experimentally.

The underlying basis for the influence on spontaneous rate of τ_Kr_ modification by ISO is shown in Figure 7. This shows the action potentials for Boyett et al. central (A) and peripheral (C) cell models (left-hand panels), and the Kurata et al. central cell (B) and peripheral (D) cell models (right-hand panels). The traces labeled “control” were obtained in the absence of ISO, the traces labeled “ISO” were obtained from simulations in which all effects of 0.05 μM ISO were considered. The traces labeled “τ_Kr_ affected only” were obtained from simulations in which only the effect of 0.05 μM ISO on “τ_Kr_” was considered. Also shown in the figure, in the lower panels, are the corresponding *I*_Kr_ records. In the Boyett et al. model simulations, 0.05 μM ISO increased the pacemaking rate by 20 and 15% for the central and peripheral cell models, respectively. In both models, ISO increased the maximal diastolic potential (i.e., become more negative; see Table 1). However, when only τ_Kr_ was affected, ISO increased the rate by 33 and 10% for the central and peripheral cell models, respectively, each with an elevated maximal diastolic potential (see Table 1). Compared with the “control” condition, in both central and peripheral cell models ISO increased *I*_Kr_ during the initial repolarization phase, but reduced *I*_Kr_ in later repolarization and the depolarization phase. These changes in *I*_Kr_ result from altered τ_Kr_. When τ_Kr_ only was affected, an increase of *I*_Kr_ in the early repolarization phase was observed, whilst a decrease of *I*_Kr_ in late repolarization period and subsequent depolarization phase was seen. The decreased *I*_Kr_ during in the depolarization phase contributed significantly to the increase of the pacemaking rate. Similar observations were seen with the Kurata et al. models (Table 1). 0.05 μM ISO increased the pacemaking rate by 25% for the central cell model and 7% for the peripheral cell model. When τ_Kr_ only was affected, ISO increased the rate by 20% for the central cell model and by 9% for the peripheral cell model.

**Table 1:** **Effects of ISO and ISO-induced change in the deactivation rate of *I*_Kr_ on the characteristics of pacemaking action potentials**.

		Boyett et al. model		Kurata et al. model	
		Central	Peripheral	Central	Peripheral
ISO (0.05 mM); all actions considered	ΔRate	20%	15%	25%	7%
	ΔMDP	−10 mV	−2 mV	−6 mV	−2 mV
ISO (0.05 mM); action on τ_Kr_ alone considered	ΔRate	33%	10%	20%	9%
	ΔMDP	+22 mV	+4 mV	+9 mV	+2 mV

In simulations, we also computed concentration-dependent change in spontaneous rate when the action of ISO on increasing *g*_Ks_ was omitted. As compared to the results obtained in the standard condition, omitting ISO action on *g*_Ks_ produced negligible effect on the pacemaking rate (data not shown).

In a previous experimental study, Lei et al. (2002) have shown that blocking *I*_Ks_ produced a negligible effect on spontaneous rate of rabbit SAN cells under control condition. However, with application of 10 nM ISO, blocking *I*_Ks_ produced ~10% prolongation of pacemaking cycle length. This implies that *I*_Ks_ plays a more important role in generating SAN pacemaking with ISO present than in its absence (Lei et al., 2002). This experimental observation on the role of *I*_Ks_ in the pacemaking potentials of rabbit SAN cells was partially reproduced by the model. In simulations, blocking *I*_Ks_ in the control condition produced negligible alteration to the pacemaking cycle length in both of the Boyett et al. central and peripheral cell models. With 10 nM ISO, blocking *I*_Ks_ produced an increase of ~3 and 2% in the pacemaking cycle length for the central and peripheral cell model, respectively. These simulation results are qualitatively similar to the experimental observations of Lei et al. (2002). Quantitative differences between the model simulation and the experimental data of Lei et al. (2002) on the role of *I*_Ks_ in SAN cell activity under control and ISO conditions may be due to the intrinsic limitations of the model as discussed in detail in Zhang et al. (2000), or due to limitations in simulating ISO (see Discussion).

### The role of *I*_st_

Similar to other currents investigated, the role of *I*_st_ in the positive chronotropic effect of ISO was investigated by comparing simulations of the effect of ISO that incorporated its effects on *I*_st_ with those in which this action was omitted. The computed data are shown in Figure 5 (black dashed line) and can be compared with the standard response incorporating all effects of ISO (solid black line). By removing the action of ISO on *I*_st_, the computed dose-dependent changes in the Boyett et al. central cell model were only slightly reduced compared with the standard values. In the Boyett et al. peripheral cell model, the computed concentration-dependent changes of rate were also close to the standard values. These results suggest that *I*_st_ does not play an important role in ISO-induced positive chronotropy under our simulation conditions. As there is no *I*_st_ in the Kurata et al. cell models, the role of augmented *I*_st_ in the positive chronotropic effect of ISO was not analyzed using the Kurata et al. cell models.

### The role of Ca^2+^ handling and Na^+^-Ca^2+^ exchange

**Figure 8:**
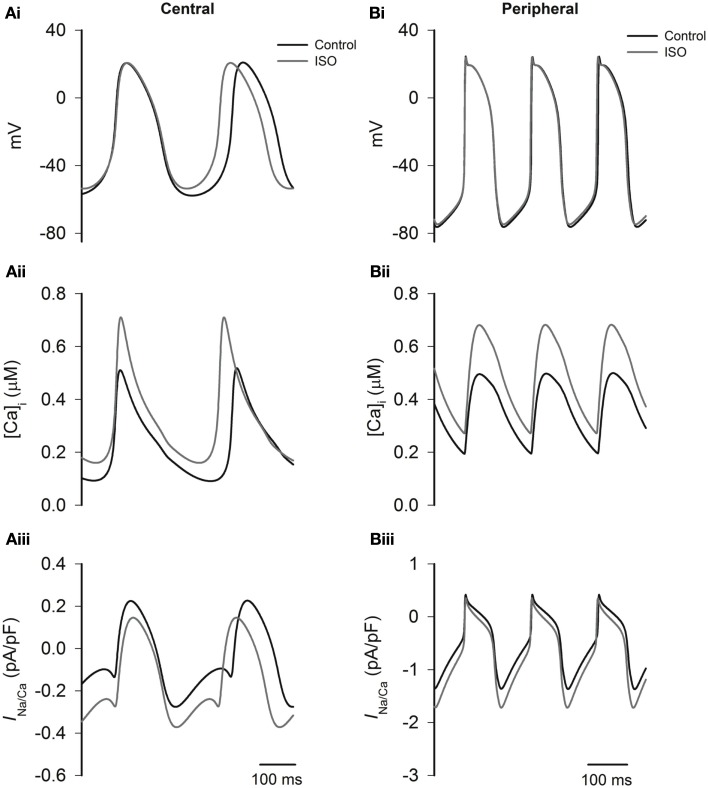
**Effects of ISO-induced changes of the Ca^2+^ transient on spontaneous activity**. Left panels show central cell models, whilst right panels show peripheral cell models (Boyett et al. models) for action potentials **(Ai,Bi)**, intracellular Ca^2+^ transients **(Aii,Bii)**, and *I*_NaCa_ **(Aiii,Biii)** under control conditions and in the presence of ISO. In ISO simulations, all effects except modulation of the intracellular Ca^2+^ transient are omitted. The simulations show that the increase in the Ca^2+^ transient plays an important role in response of the central cell model to ISO.

Experimental studies have shown that that ISO increases the systolic and diastolic levels of intracellular Ca^2+^ ([Ca^2+^]_i_) (Ju and Allen, 1999; Vinogradova et al., 2002, 2008). Here we considered how ISO-induced changes in *I*_NaCa_ consequent upon ISO-induced changes in [Ca^2+^]_i_ contribute to the positive chronotropic action of ISO, when effects on other currents are excluded. The results from Boyett et al. models are shown in Figure 8. In the figure the time traces of action potentials (Figures 8Ai,Bi), [Ca^2+^]_i_ (Figures 8Aii,Bii), and *I*_NaCa_ (Figures 8Aiii,Biii) were superimposed in different conditions. The traces labeled “control” were obtained in the absence of ISO, the traces labeled “ISO” were obtained from simulations in which the ISO-induced changes in systolic and diastolic [Ca^2+^]_i_ alone were considered. In both the central and peripheral cell models, the systolic and diastolic levels of [Ca^2+^]_i_ were doubled by ISO. In simulations using the Boyett et al. models, changes in [Ca^2+^]_i_ increased the rate by 10% for the central model, and 1% for the peripheral cell model. The accelerated pacemaking rate was attributable to an increased *I*_NaCa_ during the diastolic pacemaking phase (Figures 8Aiii,Biii) that arose from an elevated diastolic [Ca^2+^]_i_ level due to an integral action of an increased the SR Ca^2+^ release and reduced SR Ca^2+^ uptake. Further simulations were also performed to investigate the individual role of an increased SR Ca^2+^ release and a reduced SR Ca^2+^ uptake. By either increasing the SR Ca^2+^ release alone or reducing the SR Ca^2+^ uptake alone, the diastolic [Ca^2+^]_i_ level was elevated, resulting in an increased *I*_NaCa_ leading to accelerated pacemaking rates. Results from Kurata et al. cell models were qualitatively similar to the results from the Boyett et al. models.

## Discussion

In this study, we have incorporated a set of equations of the known actions of ISO on *I*_CaL_, *I*_f_, *I*_Kr_ and *I*_Ks_, *I*_st_, and intracellular Ca^2+^ handling into two established action potential models of central and peripheral rabbit SAN cells to simulate the chronotropic effect of ISO. Use of these models has enabled us to manipulate the actions of ISO and dissect out the relative contributions of different ionic currents to the modulatory actions of β-adrenergic stimulation.

The simulated effect of ISO on the spontaneous activity in the SAN cell models studied here is very similar to that seen experimentally in rabbit SAN cells (Zaza et al., 1996; Lei et al., 2000, 2002). ISO accelerated the pacemaking rate and affected the shape of action potentials in both models used here. The computed concentration-dependent increases of the rate are comparable to experimental data obtained from rabbit SAN (Zaza et al., 1996; Lei et al., 2000). With both Boyett et al. (2001) and Kurata et al. (2008) models, ISO increases the overshoot and maximal diastolic potential, and shortens the duration of action potentials. These changes in action potential shape are consistent with experimental observations (Zaza et al., 1996; Lei et al., 2000).

In our simulations, we were able to show that an increase in *I*_CaL_ contributes to the increase of the overshoot of action potential with ISO, however it contributes very little to the acceleration of firing rate. In the peripheral cell model, when the action of increasing *I*_CaL_ alone is considered, the firing rate was actually slowed down. Consistent with this, blocking *I*_CaL_ by nifedipine has been found to increase the pacemaking rate experimentally in small balls cut from the periphery of the rabbit SAN (Kodama et al., 1997). Presumably, the underlying mechanism is related to the effect of increased *I*_CaL_ on action potential duration, whereby an increase of *I*_CaL_ leads to an increase in the time interval between two successive action potentials.

The role of *I*_f_ in the chronotropic effect of ISO has been controversial. *I*_f_ has been considered by some as the main pacemaking current in rabbit SAN (DiFrancesco, 1995, 2010; DiFrancesco and Borer, 2007) and increased *I*_f_ has been considered as the main ionic basis of the chronotropic effect of ISO (Zaza et al., 1996; Liao et al., 2010). However, the role of *I*_f_ in β-adrenoceptor mediated chronotropy in the SAN is not unequivocal: Cai et al. (1995) have shown that the positive chronotropic effect of ISO was not significantly affected when *I*_f_ is blocked by Cs^+^. Also, in the amphibian sinus venosus, in which there is no *I*_f_, ISO still accelerated the firing rate (Ju and Allen, 1999), whilst mice lacking the predominant *I*_f_ channel gene (HCN4) exhibit physiological heart rate responses to isoproterenol (Herrmann et al., 2007). In our simulations, we have observed that *I*_f_ does contribute to the chronotropic effect of ISO but that its contribution is small, as removal of the action of ISO on *I*_f_ produces a small alteration to the simulated concentration-response relation for the rate increase.

The role of *I*_st_ in the chronotropy was also considered. In both models removal of the action of ISO on *I*_st_ does not affect the dose-dependent increase of rate significantly. In simulations, removal of the action of ISO had a relatively larger effect on the chronotropy in the central cell model than in the peripheral cell model. This is because in the development of the models, due to the absence of experimental data about the regional difference of *I*_st_ in rabbit SAN, the same magnitude (current density) of *I*_st_ was assumed for both central and peripheral cell models. Consequently the central cell models were relatively more sensitive to *I*_st_, due to relatively smaller current densities of other depolarizing channel currents, such as *I*_f_, *I*_Na_, and *I*_CaT_, when compared to peripheral cell models (Zhang et al., 2000).

In this study, we have shown that an increased [Ca^2+^]_i_ level in the simulated presence of ISO contributed to the increase in rate by reducing the pacemaking cycle length in the central cell model. This observation is qualitatively similar to the results of Maltsev and Lakatta (2009), which provide evidence that the “Ca^2+^ clock” plays an important role in generating sinoatrial nodal pacemaking. Quantitative differences between our results and those of Maltsev and Lakatta (2009) on the “Ca^2+^ clock” contribution to pacemaking are likely to reflect differences in the paramaterization of the various currents involved in the membrane clock between the Boyett et al. (2001) model and the Maltsev and Lakatta (2009) model. The parameters for ionic channel currents in the Boyett et al. (2001) are inherited from the Zhang et al. (2000) cell models, which were based on and validated against experimental data (Zhang et al., 2000). The difference in the response to raised [Ca^2+^]_i_ between the central and peripheral Boyett et al. (2001) models also reflected the experimentally observed intrinsic difference in the current densities of some depolarizing currents between two different cell types, as summarized in Boyett et al. (2000) and Zhang et al. (2000).

The chronotropic effect of ISO reflects the combined actions of ISO on ionic currents *I*_CaL_, *I*_f_, *I*_Kr_, *I*_Ks_, and *I*_st_, among which action on *I*_Kr_ plays a relatively more important role. Removal of the action of ISO on the rate of deactivation of *I*_Kr_ reduced the chronotropic effect of ISO significantly in both Boyett et al. (2001) and Kurata et al. (2008) models, and even reverses the chrontropy of ISO with high concentration in the peripheral model. An increase in the rate of deactivation of *I*_Kr_ increased the magnitude of *I*_Kr_ in the early action potential repolarization phase, but decreased the magnitude of *I*_Kr_ in the late repolarization phase and the depolarization phase. A decrease in outward *I*_Kr_ in the depolarization period enables the net inward current to be relatively larger, which speeds up the depolarization and thus the pacemaking rate.

The important contribution of *I*_Kr_ to cardiac pacemaking activities has been noted in previous experimental studies. In their work Sato et al. (2000) showed that blocking *I*_Kr_ partially by ibutilide (though ibutilide is not a completely selective *I*_Kr_ channel blocker) slowed down rabbit SAN pacemaking rate modestly, but blocking *I*_Kr_ completely at a higher concentration abolished its pacemaking action potentials. Ono and Ito (1995) also noted the important role of E−4031 sensitive *I*_Kr_ in rabbit SAN cardiac pacemaking. Further studies demonstrated that partial block of *I*_Kr_ (around 50%) by E−4031 almost abolished spontaneous pacemaking activity in central rabbit SAN tissue, though the pacemaking activity in peripheral SAN tissue persisted (Kodama et al., 1999). Data from mouse SAN cells also showed an important contribution of *I*_Kr_ to cardiac pacemaking action potentials as block *I*_Kr_ by E−4031 prolonged the pacemaking cycle length by about 68% (Nikmaram et al., 2008). Our simulation data added to these experimental data in showing an important role of *I*_Kr_ in SAN normal pacemaking, especially in elucidating the role of altered deactivation rate of *I*_Kr_ in cardiac pacemaking action potentials, which it is not easily possible to get from a pharmacological study.

### Assumptions, limitations, and conclusion

In the present study, due to the lack of available experimental data, we assumed that the concentration-dependent action of ISO on the current amplitude of sinoatrial *I*_Ks_ and *I*_st_ was the same as that of *I*_Kr_. This assumption requires to be re-evaluated when more experimental data become available. In addition, where data were not available from rabbit SAN cells, data from other species were used (e.g., some equation parameters for *I*_st_ were based on multiple data sources from guinea-pig and rat SAN cells (Guo et al., 1995, 1997; Shinagawa and Noma, 2000; Toyoda et al., 2005). Intracellular Ca^2+^ cycling was found to modulate pacemaking rate in the present model, but not as prominently as in a prior simulation study of Maltsev and Lakatta (2009). As the role of the Ca^2+^ clock in SAN pacemaking is debated (e.g., Honjo et al., 2003; Lancaster et al., 2004; DiFrancesco and Borer, 2007; Herrmann et al., 2007; Joung et al., 2009; DiFrancesco, 2010; Gao et al., 2010; Himeno et al., 2011; Sosunov and Anyukhovsky, 2012), further experimental quantification of SAN cellular intracellular Ca^2+^ handling mechanisms and their contribution to electrogenesis would be useful. Also due to the limited availability of experimental data on rabbit SAN cells, simulations of concentration-dependent ISO action on *I*_f_ were based on the experimental data of Zaza et al. (1996), which showed a maximal shift of the steady-state activation curve by +9.62 mV. Though this is relatively smaller as compared to the ISO-induced shift (about +7 to +18 mV) of the *I*_f_ activation curve in murine SAN cells (Alig et al., 2009; Baruscotti et al., 2010; Liao et al., 2010), it is close to observed values of 8.8 mV (Accili et al., 1997a) and 5.3 mV (Accili et al., 1997b) for a 1 μM ISO-induced shift in voltage-dependent activation of *I*_f_ in adult rabbit SAN cells. As there are no experimental data to show other ISO-induced changes in the kinetics of *I*_f_, in simulations, the slope of the steady-state activation curve and the voltage-dependent time constant of activation process were assumed to be unchanged in the ISO condition.

Whilst it is important that potential limitations of the models used in this investigation are made explicit, it is still likely that the findings of our study are valid in showing that the chronotropic effect of ISO involves an integrated action of ISO on *I*_CaL_ *I*_f_, *I*_st_, *I*_Kr_, *I*_Ks_, and [Ca^2+^]_i_) in order to match prior experimental data of Zaza et al. (1996) and Lei et al. (2000). Our study is significant in highlighting the relative contributions of *I*_f_, Ca^2+^ handling, and modulation of *I*_Kr_ deactivation kinetics to the overall response to ISO.

## Conflict of Interest Statement

The authors declare that the research was conducted in the absence of any commercial or financial relationships that could be construed as a potential conflict of interest.
